# Minimal Clinically Important Difference and Patient Acceptable Symptom State for the Pittsburgh Sleep Quality Index in Patients Who Underwent Rotator Cuff Tear Repair

**DOI:** 10.3390/ijerph18168666

**Published:** 2021-08-17

**Authors:** Umile Giuseppe Longo, Alessandra Berton, Sergio De Salvatore, Ilaria Piergentili, Erica Casciani, Aurora Faldetta, Maria Grazia De Marinis, Vincenzo Denaro

**Affiliations:** 1 Department of Orthopaedic and Trauma Surgery, Campus Bio-Medico University, Via Alvaro del Portillo, 200, Trigoria, 00128 Rome, Italy; a.berton@unicampus.it (A.B.); s.desalvatore@unicampus.it (S.D.S.); ilaria.piergentili94@gmail.com (I.P.); denaro@unicampus.it (V.D.); 2 Research Unit Nursing Science, Campus Bio-Medico di Roma University, 00128 Rome, Italy; casciani15@gmail.com (E.C.); faldetta.cbm@gmail.com (A.F.); m.demarinis@unicampus.it (M.G.D.M.)

**Keywords:** rotator cuff repair, Pittsburgh Sleep Quality Index, PSQI, minimal clinically important difference, MCID, patient acceptable symptom state, PASS

## Abstract

The Pittsburgh Sleep Quality Index (PSQI) is a valid patient-reported outcome measure developed to assess sleep quality and disturbances in clinical populations. This study aimed to calculate the minimum clinically important difference (MCID) and the patient acceptable symptom state (PASS) for the PSQI in patients who underwent rotator cuff repair (RCR). Preoperative and six-month postoperative follow-up questionnaires were completed by 50 patients (25 males and 25 females, mean age 58.7 ± 11.1 years). The MCID of the PSQI was calculated using distribution-based and anchor methods. To calculate the PSQI’s PASS, the 75th percentile approach and the receiver operating characteristic (ROC) curve were used. The MCID from preoperative to 6 months postoperative follow-up is 4.4. Patients who improved their PSQI score of 4.4 from baseline to 6 months follow-up had a clinically significant increase in their health status. The PASS is 5.5 for PSQI; therefore, a value of PSQI at least 5.5 at six months follow-up indicates that the symptom state can be considered acceptable by most patients.

## 1. Introduction

Rotator cuff tear (RCT) is the cause of 70% of all outpatient visits for shoulder pain [1]. The incidence of RCT in patients aged 60–80 years ranged from 20% to 54% [2]. Patients aged under 60 years show an incidence of 6% [3]. The main surgical techniques include open or arthroscopic techniques, suture anchor repairs, transosseous repair, transosseous tunnel technique, suture bridges, and transosseous-equivalent [4,5].

In RCT patients, sleep disturbances represent one of the leading causes that address the patient to the surgery. More than 87% of patients with RCT suffer from sleep disturbance in the preoperative period [6]. The burden of this condition is relevant, as sleeping influences biological functions [7,8], learning, memory, and quality of life [9].

However, excluding the surgery, other factors could influence the satisfaction of the patients and the quality of sleep after RCR. Maestroni et al. [10] reported that the etiology of sleep disturbance in patients with RCT could be multifactorial due to age, sex, comorbidities, and external conditions. Khazzam et al. [9] confirmed this hypothesis, adding cervical conditions, diabetes mellitus, and obesity as co-leading causes of sleeping disturbance in RCT patients. Gumina et al. described a relationship between rotator cuff tear size and a higher value of VAS pain and sleep disturbances [1]. To our knowledge, although sleep quality in patients with RCT is frequent, few studies investigated possible sleep improvement after surgery. Finding valid patient-reported outcome measures (PROMs) to assess this outcome could be interesting for international researchers.

PROMs are self-reported patient measures that aim to report specific outcomes, avoiding third party interpretation [11]. PROMs could be general, evaluating pain, functionality, quality of life, and sleep, or specific (e.g., joint awareness after prosthetic replacement) [12]. First described in 1988, the Pittsburgh Sleep Quality Index (PSQI) is an example of a PROM [13]. This score was developed to assess the sleep quality and disturbances in clinical populations. The PSQI is a 19-item self-report tool created to assess sleep quality. The PSQI score has a possible range of 0–21 points, and high scores are related to poor sleep quality.

Different scores are widely used in clinical practice, but they should be easily interpretable to provide valid information to researchers. There are different methods to facilitate the interpretation of the PROMs. One of the most commonly adopted is the minimally clinical important difference (MCID) [14] and the most recent tool, patient acceptable symptom state (PASS). The utility of clinical practice is usually assessed by the difference between preoperative and postoperative PROMs. While this variation can reflect a change in sleep quality following RCT repair, it does not indicate the magnitude of the effect size [15]. A clinically significant mean change should not reflect a real change for the patient [14]. MCID is the difference in PSQI scores between patients with no sleep quality changes and patients with “small” improvements after RCR. The term MCID is often confused or interchangeably used with the minimum important change (MIC) [15].

Instead, the minimum PROM threshold that correlates to a patient’s satisfactory state [16] is named a PASS. MCID and PASS are complementary concepts. They are both based on an external anchor question, but while MCID assesses improvement (feeling better), the PASS evaluates the satisfactory final state of the patient (feeling good). However, MCID is a tool that cannot be trusted if assessed in only one study or by one method. Therefore, it is necessary to provide multiple studies or a different method to evaluate the MCID.

The PSQI was recognized as a valid and reliable PROM to assess sleep quality [13]. However, to our knowledge, the MCID and PASS of this questionnaire have not already been determined in RCT patients.

This study aimed to estimate the value of PSQI in patients who received RCR and perceive the procedure as successful. To address this issue, both distribution-based and anchor methods were used to assess the MCID and PASS of PSQI.

## 2. Materials and Methods

This is a quality improvement study. From February 2019 to June 2020, 66 participating patients underwent arthroscopic rotator cuff repair. The senior surgeon assessed all the patients with a clinical examination (specialized in shoulder arthroscopy) and confirmed the diagnosis of RCT by preoperative magnetic resonance imaging (MRI). Inclusion criteria were: patients with Goutallier grade 2 [17] and Patte stage 2 lesions [17], previously treated conservatively (with physical therapy and corticosteroid injections). Both single- and double-bundle techniques were used to repair RCTs [18,19]. The same senior surgeon performed all the procedures. Patients who did not undergo surgery or with other types of shoulder pathologies were excluded. A standardized rehabilitation protocol was prescribed after surgery to all the patients included [20].

All of the eligible patients completed preoperative surveys and agreed to enroll in this prospective study. Fifty patients (25 males and 25 females, mean age 58.7 ± 11.1 years) completed the 6-month follow-up and were included in the analysis. All patients included completed the PSQI preoperatively and at six months postoperatively.

The PSQI is a 19-item self-report tool for determining sleep quality. The PSQI score may range from 0 to 21, with high scores indicating clinically significant sleep problems. The PSQI questionnaire was translated and validated in the Italian language by Curcio et al. [21]. The Italian version of PSQI was reliable with a high internal consistency (Cronbach’s a = 0.835) [21].

### 2.1. Statistical Analysis

The minimum number of patients expected was 34, based on a 0.5 Cohen’s d effect size obtained from the difference in PSQI between preoperative and six months postoperative follow-up in patients with rotator cuff disease [22], a power of 80%, and a type 1 error of 0.05 (two-tailed). The Shapiro-Wilk test was used to determine data normality. The paired T-test was used to assess baseline and six-month postoperative scores as the data reported a standard distribution. The statistical significance level was set at *p* < 0.05. SPSS version 26 was used to analyze all results (Armonk, NY: IBM Corp).

#### 2.1.1. Calculation of MCID

The 0.5 SD (0.5 standard deviations), SEM (standard error of measurement), and the MDC (minimum detectable change) were used as distribution-based approaches [23,24]. A medium effect size was correlated with the 0.5 SD [23,24]. The SEM denotes the smallest variation above the measurement error (ME) [23,24]. With a 95% confidence interval, the MDC indicates the smallest change above the measurement error [23,24].

For this analysis, Cronbach’s alpha was used to measure the reliability of the PSQI when calculating the SEM and MDC [24,25]. At the 6-month follow-up, the following question was asked to the patients “How do you feel after the surgery performed?” with “Much Worse”, “A Little Worse”, “Equal”, “A Little Better”, and “Much Better” as a possible answer. Patients who responded “Much Worse”, “A Little Worse”, or “Equal” were considered non-responders, while patients who responded “A Little Better” were considered minimally improved [24]. This question was used as an anchor. The improvement in PSQI cut-off was identified using receiver operating characteristic (ROC) curves with the maximized sensitivity and specificity (with Youden index) [24]. The area under the curve (AUC) was determined using the ROC technique, which measures a questionnaire’s ability to identify patients who have changed from those who have not, based on external criteria. A test with an AUC of 1.0 has perfect discriminatory ability (100 percent sensitivity and 100 percent specificity). A test with an AUC of 0.5 is said to have no discriminating value. According to Terwee et al., a valid anchor should have a criteria value of 0.7 or above [26]. The MCID was also estimated using the mean change (MC) in PSQI, the change in score for patients who reported a minimal improvement, i.e., the patients who responded: “A Little Better” [24].

#### 2.1.2. Calculation of PASS

A valid anchor for PASS needs to consider the pain, physical function, and satisfaction of patients [27]. Kvien et al., as an anchor for PASS, suggested the following question “Taking into account all the activities you have during your daily life, your level of pain, and also your functional impairment, do you consider that your current state is satisfactory?” [27]. For the present study, to calculate PSQI’s PASS, the question “In general, would you say that your health is at least good?” was used. The possible answers were “Yes” or “No”. Patients who responded “Yes” were considered in an acceptable state of symptoms. PASS thresholds of PSQI were measured using the 75th percentile of the cumulative percentage curve of patients who consider themselves in an acceptable state of symptoms and the point on the receiver operating characteristic (ROC) curve, in which the cut-off was measured using the Youden index [27,28,29].

## 3. Results

This study included 50 patients (25 males and 25 females, mean age 58.7 ± 11.1 years) treated by RCR. All of them completed the 6-month follow-up and were included in the analysis. In addition, all the patients answered the PSQI questionnaires both before and six months after surgery. The PSQI change score was considered normally distributed using Shapiro-Wilk tests of normality (*p* = 0.129).

The baseline PSQI mean score was 7.5 ± 4.1 (with a range between 2 and 18). The 6-months postoperative PSQI mean score was 3.9 ± 3.5 (with a range between 0 and 14). There was a statistically significant difference in the PSQI score between baseline and the six-month follow-up (*p* < 0.001).

MCID estimates for the PSQI index score ranged from 1.3 to 4.4. Depending on the type of calculation approach, different MCID values were estimated.

**Figure 1:**
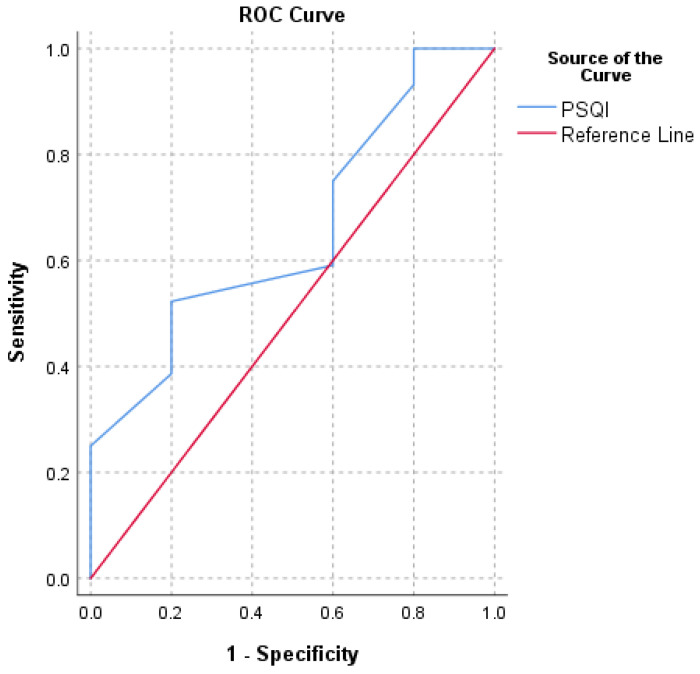
Receiver operating characteristic curve (ROC) for the prediction of PSQI’s minimum clinically important difference (MCID) based on the question “How do you feel after the surgery performed?”.

An MCID of 1.4 was discovered using the 0.5 SD method. The SEM approach was used to generate an MCID of 1.3 (with good internal consistency reliability, Cronbach’s α = 0.8). An MCID of 3.5 was found using the MDC method (at a 95% confidence level). An MCID value of 1.5 was calculated using the ROC technique (with a high instrument of responsiveness, AUC = 0.8) (Figure 1). The mean change (MC) method yielded an MCID of 4.4. (Table 1).

**Figure 2:**
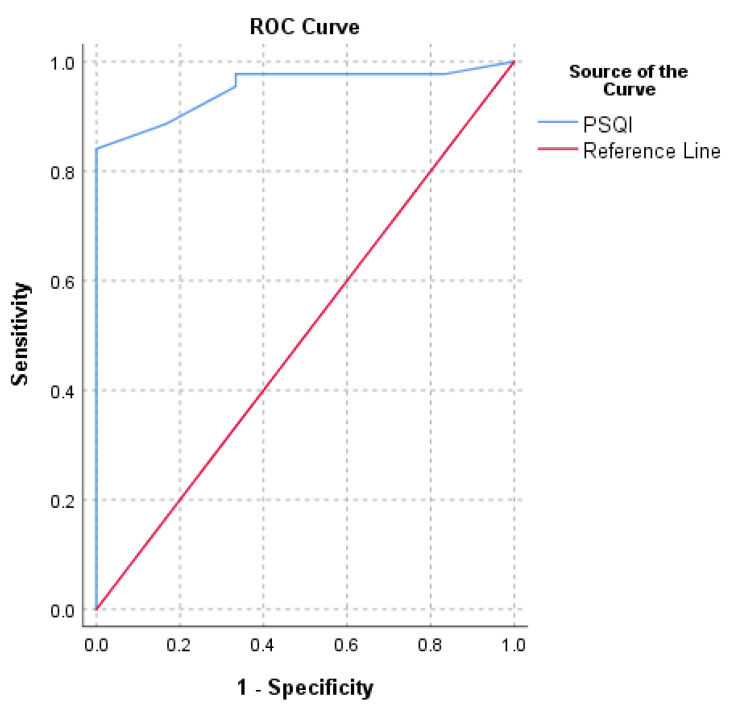
Receiver operating characteristic curve (ROC) for the prediction of PSQI’s patient acceptable symptom state (PASS) based on the question “In general, would you say that your health is at least good?”.

PASS calculated for PSQI were 4 and 5.5. The PSQI value for identifying a PASS that maximized sensitivity and specificity (with the ROC method) was 5.5 (AUC = 0.9) (Figure 2). The cut-off value computed with the 75th percentile approach was 4 (Table 2).

## 4. Discussion

The study aimed to determine the PSQI MCID and PASS in 50 patients who received RCR with a 6-month follow-up period. In the literature, several studies report an MCID of PSQI ≥ 3 [30,31,32,33]. Two meta-analysis report values for PSQI’s MCID between 1.54 and 3 [34,35]. However, none of these studies calculated the MCID of the PSQI for patients who underwent RCR.

Jaeschke et al. coined the term MCID to describe the smallest variation in score in the domain of interest that patients consider as beneficial [36]. As a result, the MCID is a quantifiable minimal threshold value in a score of interest that patients interpret as an improvement in their health [37]. Several strategies, divided into distribution-based approaches and anchor approaches, may be used to determine the MCID. The 0.5 SD method [23,24], SEM [23,24], and MDC [23,24] are examples of the former, which are based on the statistical properties of a study’s results [38]. A clinically significant change with a medium effect size [39] is represented by the 0.5 SD method. The SEM represents the variation caused by the unreliability of the scale or measurement errors [40]. A valid MCID, according to Copay et al., should be at least larger than the SEM value and correlate to the patient’s perception of the change [41]. Furthermore, a beneficial MCID should be larger than the MDC, according to Stipancic et al. [42]. With a 95% level of confidence, the MDC is the smallest change that can be considered above the measurement error.

In addition, in orthopedic investigations, anchoring approaches are most widely utilized [43,44,45]. Based on these two criteria, the ROC and MC methods [24] were the most suitable. However, the MCID calculation with the ROC method was not helpful because it was less than MDC. Therefore, the MC approach seems to be the most appropriate. For these reasons, the MCID of PSQI for patients who underwent RCR was 4.4. To summarize, a change greater than 3.5 (the MDC) indicates that the change is unlikely to be due to chance variability. In comparison, a change greater than 4.4 (the MCID) indicates that this change is clinically meaningful.

The second purpose of this study was to determine the PSQI PASS six months after RCR. The PASS threshold on a PROM is the value most closely linked to patient satisfaction, assessed by a different questionnaire. Using both the 75th percentile and ROC approaches, two PASS values were found following the anchor “In general, would you say your health is at least good?”. The PASS cut-offs were 5.5 (AUC = 0.9) with the ROC approach and 4 with the 75th percentile approach. Since a high value of AUC was identified, the ROC method seems to be the most appropriate. Moreover, the value found with the ROC method is closest to 5, the threshold for insomnia in PSQI [13,46,47,48]. Therefore, the PASS value of PSQI for patients who underwent RCR was 5.5.

### Strengths and Limitations

Longo and colleagues assessed different dimensions of sleep quality using the PSQI, in a sample of 58 consecutive patients undergoing RCR surgery [1]. However, as demonstrated by several authors, a statistically significant improvement in a score does not ever reflect an improvement in a patient’s perception of clinical benefit. MCID and PASS could solve this limitation. To our knowledge, this is the first study that has investigated the MCID and PASS of PSQI in RCR patients. Moreover, the MCID and PASS were calculated using the most popular ad hoc methods. Furthermore, MCID was measured using both the distribution and anchor methods. Finally, the sample size is larger than the power analyses’ minimum number of patients. The burden of RCT and RCR surgery is relevant for the healthcare system. Consequently, it is essential to evaluate the most common conditions associated with RCT, like insomnia. Furthermore, finding new tools to assess the real perception of clinical benefit for the patient could be helpful for clinicians to estimate the real influence of surgery. Therefore, calculation of the MCID and PASS of PSQI could be interesting for the international community.

However, this article has some weaknesses. First, the MCID and PASS were estimated for a six-month follow-up and cannot provide information for a longer period. Long-term follow-up can reveal differences in MCID values. Furthermore, since only one anchor was used, the consistency of findings across anchors was not evaluated. Lastly, patients may suffer from insomnia as a chronic disorder or may receive treatment in the six months between surgery and reassessment. Moreover, some confounders as comorbidities, such as sleep disorder breathing, obesity, and drugs abuse, were not considered. Therefore, there are many factors that could influence sleep.

## 5. Conclusions

In this study, the PSQI MCID and PASS values from 50 patients who underwent RCR were analyzed. The MCID from baseline to 6 months postoperative follow-up was 4.4. Patients who improved their PSQI score of 4.4 from baseline to the 6-month follow-up had a clinically significant increase in their health status. The PASS is 5.5 for PSQI; therefore, a value of PSQI at least 5.5 at the six-month follow-up indicates that the symptom state can be considered acceptable by most patients.

## Figures and Tables

**Table 1:** MCID for PSQI calculated by both distribution-based and anchor approaches ^1^.

Score	0.5 SD	SEM	MDC	ROC (AUC)	MC
PSQI	1.4	1.3	3.5	1.5 (0.8)	4.4

^1^ PSQI: Pittsburgh Sleep Quality Index, 0.5 SD: 0.5 Standard Deviation, SEM: Standard Error of Measurement, MDC: Minimal Detectable Change, ROC: Receiver Operating Characteristic, AUC: Area Under the Curve, MC: Mean Change.

**Table 2:** Patient acceptable symptom state (PASS) for PSQI ^1^.

Score	ROC (AUC)	75th Percentile
PSQI	5.5 (0.9)	4

^1^ PSQI: Pittsburgh Sleep Quality Index, ROC: Receiver Operating Characteristic, AUC: Area Under the Curve.

## Data Availability

The data presented in this study are available on request from the corresponding author. The data are not publicly available due to privacy.
